# Volatile organic compounds: A proinflammatory activator in autoimmune diseases

**DOI:** 10.3389/fimmu.2022.928379

**Published:** 2022-07-29

**Authors:** John Onyebuchi Ogbodo, Amarachukwu Vivan Arazu, Tochukwu Chisom Iguh, Ngozichukwuka Julie Onwodi, Tobechukwu Christian Ezike

**Affiliations:** ^1^ Department of Science Laboratory Technology, University of Nigeria, Nsukkagu, Enugu State, Nigeria; ^2^ Department of Plant Science and Biotechnology, University of Nigeria, Nsukka, Enugu State, Nigeria; ^3^ Department of Pharmaceutical Technology and Industrial Pharmacy, University of Nigeria, Nsukka, Enugu State, Nigeria; ^4^ Department of Biochemistry, University of Nigeria, Nsukka, Enugu State, Nigeria

**Keywords:** VOC, proinflammatory mediators, autoimmune diseases, immune system and etiopathogenesis

## Abstract

The etiopathogenesis of inflammatory and autoimmune diseases, including pulmonary disease, atherosclerosis, and rheumatoid arthritis, has been linked to human exposure to volatile organic compounds (VOC) present in the environment. Chronic inflammation due to immune breakdown and malfunctioning of the immune system has been projected to play a major role in the initiation and progression of autoimmune disorders. Macrophages, major phagocytes involved in the regulation of chronic inflammation, are a major target of VOC. Excessive and prolonged activation of immune cells (T and B lymphocytes) and overexpression of the master pro-inflammatory constituents [cytokine and tumor necrosis factor-alpha, together with other mediators (interleukin-6, interleukin-1, and interferon-gamma)] have been shown to play a central role in the pathogenesis of autoimmune inflammatory responses. The function and efficiency of the immune system resulting in immunostimulation and immunosuppression are a result of exogenous and endogenous factors. An autoimmune disorder is a by-product of the overproduction of these inflammatory mediators. Additionally, an excess of these toxicants helps in promoting autoimmunity through alterations in DNA methylation in CD4 T cells. The purpose of this review is to shed light on the possible role of VOC exposure in the onset and progression of autoimmune diseases.

## Introduction

The presence of volatile organic compounds (VOCs) in our environments is a result of both human activities and natural factors (1). VOCs are a large group of organic chemical compounds found in many day-to-day products that can vaporize easily when exposed. They are characterized by high volatility and mobility with strong resistances to abiotic and biotic degradation (2). VOCs are widely emitted from paint during automobile spray, paint industries, varnishes, waxes, and solvents used in furniture making and electronic machines (3). They are also factors of nature discharge during a volcanic eruption (4). VOCs are organic compounds with many functional groups, such as toluene, formaldehyde, xylene, and benzene used in paint formulation (5). The negative effect of VOCs can be expressed in both abiotic and biotic systems (6). It is a major source of environmental pollution (7). VOCs produce hazardous environmental pollutants by interacting with other chemical compounds such as nitrogen oxide (NO*_x_*) to form tropospheric ozone (O_3_), a primary pollutant, and a secondary pollutant, such as peroxyacetyl nitrate, which has more serious health and environmental effects (8). The effects of VOCs on the human system is attained through a health risk assessment, which is one of the major means of determining the short- and long-term effects on people exposed to VOCs (9).

Volatile organic compounds enter the human system through three major routes, such as lungs (inhalation), dermal contact (absorption), and mouth (ingestion) (10). The accumulative effect of the inhalation of VOCs causes different adverse health effects starting from the epithelial lining of the respiratory tract and mucous membrane (10). The negative effect of VOCs is occupationally linked due to long-term exposure leading to nausea, anxiety, headache and chronic conditions such as damage to the liver, skin, and respiratory and nervous systems (11). Chemical compounds containing substances such as propylene glycol (PG) and glycol ethers, formaldehyde, and benzene react actively with the component (mucin) of the mucous membrane of the epithelial lining of the respiratory system. This is because mucin is a polymer of glycoproteins, which is rich in amine groups. This amino end of mucin is nucleophilic, reacting with C–O polar bonds in VOCs. The long-term exposure of an organism to VOCs results in a series of intramolecular reactions leading to the formation of a double-bonded C–N group, cross-linking with other similar compounds in the mucin. An antibody, immunoglobin E (IgE) detects these antigens (chemical reactions as a result of exposure) and triggers an inflammatory response. Mucin is also found in the eyes and esophageal lining, leading to irritation and dryness of the skin (12).

The cell, tissue, or organ affected by volatile organic compounds depends on the level of exposure, whether acute or chronic. The most affected system in acute exposure to VOCs is the respiratory and the central nervous systems, causing headache, dizziness, and irritation of the eyes, nose, throat, and membranes, such as the mucin membrane (13). Chronic exposure due to occupation or family settlement raises much concern to the effect as a result of acute exposure. This type of exposure (chronic) affects different parts of the living organism such as the immune, hematopoietic, respiratory, and central nervous systems and organs such as the liver (the hub of metabolism) and kidney (14). This chronic effect of VOCs causes immunodeficiency, which alters blood chemistry, resulting in leukemia, slow biochemical reactions, hormonal and electrolyte imbalance, memory loss, asthma, peripheral neuropathy, and other symptoms. Furthermore, because VOCs are lipid soluble, they can cross the blood–brain barrier and affect the neurological system (15).

## Categories of VOCs

Based on chemical composition and physical properties, VOCs are described as organic compounds that consist of elements of carbon and one or more functional groups that include elements such as oxygen, phosphorus, nitrogen, silicon, halogen, and sulfur. The United States Environmental Protection Agency defined VOCs as compounds of carbon excluding carbon dioxide, carbon monoxide, carbonic acids, ammonium carbonate, metallic carbonates, and carbides that trigger photochemical reactions in the atmosphere (16). Under average physical conditions of temperature and pressure, VOCs are characterized by a low boiling point, high saturation vapor pressure, and high volatility (17). They are often classified into three categories based on their boiling point and the ease with which they are emitted. With respect to boiling point, VOCs are categorized as very volatile organic compounds (VVOCs), volatile organic compounds (VOCs), and semi-volatile organic compounds (SVOCs) (18). VVOCs have a boiling point ranging from 0 to 50–100°C, while VOCs include organic compounds with a boiling point ranging from 50–100 to 240–260°C, and SVOCs have a boiling point ranging from 240–260 to 380–400°C (17).

Volatile organic compounds constitute organic compounds with boiling points within the range of 50–100 to 240–260°C. At 293.15 K, they possess a vapor pressure of 0.01 KPa. There are numerous compounds categorized under volatile organic compounds, and they include saturated and unsaturated linear hydrocarbons (*e*.*g*., heptane, hexane, mineral spirits, and pentane), aromatic hydrocarbons such as alcohols, aliphatic hydrocarbons, ketones, aldehydes, ethers acids, and chlorinated compounds (such as chlorofluorocarbons, dichloromethane, trichloroethylene, and tetrachloroethylene), nitrogen compounds (amines and nitriles), sulphur compounds (dimethyl sulfide and thiols), and plasticizers such as dicotyl and phthalate (18). Other identified VOCs include terpenes (hemiterpenoids, monoterpenoids, and sesquiterpenoids), organic compounds with heteroatoms (*e*.*g*., S, N, Cl, and Br), organosulfates, organonitrates, organohalides, polychlorinated biphenyls (PCBs), and polycyclic aromatic hydrocarbons (PAHs) (19). Characteristically, VOCs are inert lipophilic compounds and possess the ability to permeate biological membranes (20). They also have low water solubility and the least vapor pressure of 0.01l Pa at 20°C (21).

Organic compounds that are more volatile than the VOCs are classified as VVOCs. VVOCs are characterized by a lower boiling point of the range <0 to 50–100°C (22). The development of ISO 16000-6 provided an analytical-based definition of VVOCs to include organic compounds which elute between n-hexane and n-hexadecane (C6–C16) in a non-polar or slightly polar gas chromatographic separation column (23, 24). More precisely, VVOCs are separated in a gas column chromatography on a 5% phenyl/95% methyl-polysiloxane column before n-hexane with a retention index of less than 600. VVOCs are also categorized according to their vapor pressure, which includes compounds with vapor pressures greater than 100 Pa and/or greater than 1,000 Pa and distinguishes between volatile organic compounds and semi-volatile organic compounds (23). On the other hand, there is no upper limit designating a boundary between VVOCs and VOCs (24). According to Salthammer (23), there is unsubstantial knowledge and information on the VVOC subgroup of indoor pollutants and there are no routinely analytical methods designated for analyzing VVOCs. However, gas chromatography, liquid chromatographic methods, and online methods (photoacoustic spectroscopy, photoionization detector, and proton transfer reaction mass spectrometry) are analytical methods that can be utilized in monitoring and analyzing VVOCs. Commonly observed VVOCs include acetaldehyde, acrolein, 1,3-butadiene, formaldehyde, formic acid, acetic acid, methyl bromide, methyl chloride, and propane (22, 23, 25).

SVOCs include all volatile organic compounds with a higher molecular weight than VOCs (26) and a boiling point ranging from 240–260 to 380–400°C (17, 27). They have a vapor pressure between the ranges of 10^-9^ to 10 Pa at a temperature of 25°C (28, 29). Because of their lower vapor pressure, they occur in gas and condensed phases and are easily mixed into materials without the formation of chemical bonds. Therefore, they are released slowly into the environment and are absorbed as air pollutants through non-dietary dust ingestion, inhalation, and dermal absorption. SVOCs are highly toxic and can be transported to long-range distances from their site of emission. Additionally, they are difficult to remove through degradation process and are predominant in complex organic chemicals used for agricultural purposes. Examples include PCBs, polybrominated diphenyl ethers, phthalic acid esters, and PAHs (30).

Cleaning products, electronic devices, textiles, and plastic items are major sources of SVOCs found in households and indoor places. These products contain high amounts of additives such as antioxidants, flame retardants, perfluorinated compounds, and plasticizers that emit SVOCs into the environment (27). More so, uncontrolled burning, incineration of municipal wastes, and certain waste management and elimination processes result in the emission of SVOCs (30). Hexabromocyclododecanes, chlordane (C_10_H_6_C_l8_), benzyl alcohol (C_7_H_8_O), and phthalates are common SVOCs that cause atmospheric air pollution (26).

## Sources of volatile organic compounds

The emission of VOCs can be induced naturally or through anthropogenic sources. Nonetheless, anthropogenic sources are the major contributing factors to the generation and release of VOCs, especially in industrial areas (31). VOCs are released by vegetation, anaerobic marshy bog processes, and forest fires and other biogenic processes for the natural sources, while the anthropogenic sources of emission are in the form of domestic and industrial processes such as septic system, vehicular exhaust, chlorination, food extraction, fertilizer and pesticide applications, hydrocarbon fuel evaporation, printing, and pharmaceutical processes (32, 33). The sources of VOC emission are majorly identified using the receptor-oriented model and the positive matrix factorization (PMF) tool which is commonly utilized for source apportionment in identifying and designating the origin and source of VOCs. The PMF tool works by decomposing the test samples and materials into two matrices, where one matrix is designated as factor contributions (G) and the other as factor profile (F). The multivariate tool is for obtaining the source composition profiles which are used to determine the potential source of emission of the VOCs present in a test site or material (8).

## Natural sources of VOCs

Some biogenic and natural processes that occur in nature cause the formation and emission of VOCs into the atmosphere. Forest fires and vegetation fires are one of the major biogenic sources that cause environmental pollution (32). Non-methane hydrocarbons emitted by vegetation are referred to as biogenic volatile organic compounds (BVOCs). The BVOCs of most concern include monoterpenes, isoprene, and sesquiterpenes of the class isoprenoids (34). They are produced by undisturbed trees and crops in response to biotic and abiotic stresses, oxidative stress, and heat. These isoprenoids are also responsible for antiherbivory functions in plants. In (32), an elevated amount of VOCs caused by Russian summer forest fire episodes in 2006 and 2010 was recorded. The increased quantity of VOCs influenced the air in Finland with methanol, acetaldehyde, monoterpenes, and acetone recorded as VOCs with biogenic origins. Consequently, Mouat et al. (35), recorded the introduction of propane, acrolein, methyl propanoate, methyl guaiacol, maleic anhydride, methyl methacrylate, methyl benzoic acid, and pentanones to temperate Australian forest caused by the 2019/2020 Australian wildfire. Furthermore, Whitehill et al. (36) investigated the effect of prescribed pasture burning of tallgrass prairie in the Flint Hills region of Kansas state in the United State of America on the air quality and VOC concentrations of the region. The reports show emissions of high amounts of VOCs caused by vegetation fires, with 32 VOCs identified to be present above the method quantification limit. The vegetation fire emitted a negligible amount of three compounds, two of which were dichlorodifluoromethane and trichlorofluoromethane. The 29 remaining identified VOCs, on the other hand, include unsaturated compounds with alkenes (propane, n-pentane, isopentane, butane, and n-hexane) with higher emission factors. Other emitted compounds comprise propylene, a C3 compound, C4 (cis-2-butene, 1-butene, 1,3-butadiene, and trans-2-butene), C5 (isoprene, cis-2-pentene, 1-pentene trans-2-pentene), 1-hexene which is a C6 compound (1-hexene), and alkanes (propane, n-pentane, isopentane, butane, n-hexane). More so, simple nitriles such as acrylonitrile and acetonitrile and simple aromatic compounds like toluene, ethylbenzene, benzene, and p-xylene were observed with oxygenated VOCs that include ethanol, vinyl acetate, acrolein, 2-butanone, and ethanol.

In addition to forest fires, biogenic VOCs are generated through anaerobic marshy bog processes. Microorganisms release a significant amount of VOCs through soil, litter, and other microbial processes. Soil bacteria and fungi are the primary organisms responsible for the release of BVOCs from the soil, producing a wide array of VOCs through metabolic and biochemical activities (37). Leff and Fierer (37) examined microbial-induced VOCs in soil and litter samples. About 100 VOCs were identified from both soil and litter samples. The litter samples produced higher rates of VOCs than the soil samples and showed a wider range of VOC classes with compounds such as terpenoids, which were not emitted from soil samples. Other identified VOCs observed include furfural, butanoic, and propanoic acids. The volatile organic compounds with high identification quality rating greater than 95% include α-and β-pinene, camphene, tetrachloroethylene, D-limonene, o-xylene or p-xylene, benzene compounds, ethyl compounds, naphthalene, ethanol, 1,4-cyclohexadiene, and acetate (37). **Table 1** shows a summary of the different types of VOCs including their sources and exposure phenotypes.

**Table 1:** Various volatile organic compound (VOC) types and their exposure phenotypes.

Types of VOC/sources	Example	Exposure phenotype	References
Aromatic hydrocarbons: automobile exhaust fumes, gasoline evaporation, cigarette smoke, petroleum refining, a component of paint and printing ink (38)	Benzene	Alterations in gene expression associated with apoptosis, metabolic oxidative stress, and inflammatory cytokine production (mice)	(39)
	Toluene	High doses affect IFN-ϒ, IL-4, and IL-13 production by activated T cells and increase TNF-α expression (human PBMCs)	(40)
	Ethylbenzene	Associated with higher percentages of IL-4-producing CD^3+^ T cells (human)	(41)
	Xylene	Generates ROS, triggers oxidative stress, and oxidant injury (human lymphocytes)	(42)
	Styrene	Causes chromosomal aberrations, increase in monocytes, and cell adhesion molecules (mice)	(43)
Polycyclic aromatic hydrocarbons: incomplete fuel combustion, asphalt transformation plants, and coal power plants (44, 45)	Naphthalene	Triggers a considerable reduction in the number of CD3+/CD8+ peripheral T cells (human)	(41)
	Phenanthrene	Induces adaptive immune response changes (Th1/Th2-related cytokine release) (human)	(46)
	Benzo[a]pyrene	Alters the development of T lymphocytes in offspring (mice)	(47)
Aldehydes: emission from interior decorative materials, cosmetics, cleaning agents, treated wood resins, plastic adhesives (48)	Formaldehyde	Reduces the number of NK cells, regulatory T cells, and CD8^+^ effector memory T cells (human)	(49)
	Acetaldehyde	Modifies self-proteins and induces autoimmunity, resulting in increased inflammation	(50)
Chlorinated hydrocarbons: components of paints, rubbers, cleaning agents, formulation and processing of chemical extractants, drugs production (51)	Dichloromethane	Induces IFN-related genes as part of the immune response which was then followed by the apoptosis pathway (human promyelocytic leukemia HL60 cells)	(52)
	1,2-dichloroethane	Induces an overproduction of inflammatory factors in the brain (mice)	(53)
	Vinyl chloride	Its metabolites induce organ injury *via* inflammation (mice)	(54)
	Trichloroethylene	Increases CD4+ and CD8+ T cell populations (mice)	(55)
	Chlorobenzene	Associated with higher percentages of IL-4-producing CD^3+^ T cells (human)	(41)
Alcohols: cosmetics and personal care products such as nail polish, nail polish removers, colognes, perfumes, rubbing alcohol, and hair spray	Ethyl alcohol Isopropyl alcohol Benzyl alcohol	Excessive consumption decreases the number of peripheral T cells, disturbs the balance of distinct T cell types, impacts T cell activation, inhibits T cell function, and increases T cell death (human and animal model)	(56)
Ketones: aerosols, varnishes, window cleaners, paint thinners, plastics, and Adhesives	Polymethyl-methacrylate	Alters immune cell function and deters host response to antigenic stimuli (mice)	(57)
	Methyl ethyl ketone	Induces the secretion of proinflammatory cytokines (humans)	(58)

## Anthropogenic sources

Anthropogenic-generated VOCs come from both domestic products (such as solvents, perfumes, inks, gasoline, paints, dyes, cleaners, paint removers, tobacco smokes, and adhesives) and industrial and agricultural processes that include food extraction processes, metal surface degreasing, hydrocarbon fuel evaporation, vehicular exhaust, printing, building, septic system, fumigation, fertilizer, and pesticide application (33, 59). While biogenic emissions of VOCs are important globally, emissions from anthropogenic sources dominate urban cities (59, 60) and constitute majorly VOCs from traffic and the burning of biomass (31). A recent study conducted by Liu et al. (61) indicates that vehicular exhaust contributes 22–58% of VOC emission in China. However, there are differences in the sources that contribute to VOC emission across regions in China. In the cities of Langfang and Xiamen, paint solvents constitute the highest contributing factor to VOC emission (62), while vehicular exhaust and combustion are the primary sources of VOCs in Wuhan City (63). In another study (59), it was reported that about 40 VOCs were observed in more than 50% of air samples collected from residences of pregnant women in Northeastern British Columbia, a region of natural gas exploitation, while four VOCs were detected in more than 50% of the water samples tested. On the other hand, indoor air concentrations showed more than 95% of CHMS in the range of 10–60% of the tested samples. The VOCs detected in the samples include acetone, 1,4-dioxane, 2-methyl-2-propanol, decanal, chloroform, decamethylcyclopentasiloxane, hexanal, m/p-xylene, styrene, o-xylene, and dodecane. Pinthong et al. (8) investigated the source identification of VOCs in Map Ta Phut industrial complex of Rayong Province, East Thailand, using the PMF model. Comprehensive monitoring of VOCs to determine emission sources revealed that benzene, xylene, 1,2-dichloroethane, 1,2,4-trimethylbenzene vinyl chloride, and ethylbenzene 1,3,5-trimethylbenzene were the most prevalent VOCs. Gasoline combustion in vehicles releases xylene compounds, benzene, and ethybenzene 1,2-dichloroethane and vinyl chloride are the primary VOCs emitted in PVC industries (64, 65). As a result, industrial operations are the primary sources of VOCs in urban areas. The household VOCs detected were generally linked with the chemicals used in households with associated VOCs including acrylonitrile, 1,2-dichloropropene, 1,1,1-trichloroethane, 1,1-dichloroethylene, 1,2-dibromoethane, and benzyl chloride (8). According to the research conducted by Pinthong et al. (8), vehicle exhaust and industrial processes contributed more to the emission of VOCs in industrial areas. The PVC and petrochemical industries were identified as major emission sources during the wet season due to high concentrations of 1,2-dichloroethane and 1,3-butadiene, which are the major resources used in PVC and petrochemical industries, respectively. Generally, widespread industrial activities, energy consumption, busy traffic, and household chemical products contribute to the large release of VOCs in both local and urban areas (66, 67).

## Volatile organic compounds as a proinflammatory activator

The actual mechanism of how VOCs trigger inflammation in humans has not been clearly elucidated. Several *in vitro* studies utilizing rat models or cell lines, however, demonstrated that exposure to a significant amount of VOC promotes oxidative stress and stimulates the generation of inflammatory mediators in human lung epithelial cells (68). Hence, the actual mechanism of VOC toxicity is oxidative stress induction. It was thought to be the main mechanism of cigarette smoke-induced lung damage, acute aggravation of chronic obstructive pulmonary disease, and many other deleterious effects exerted by other air pollutants on the respiratory epithelium (69).

**Figure 1:**
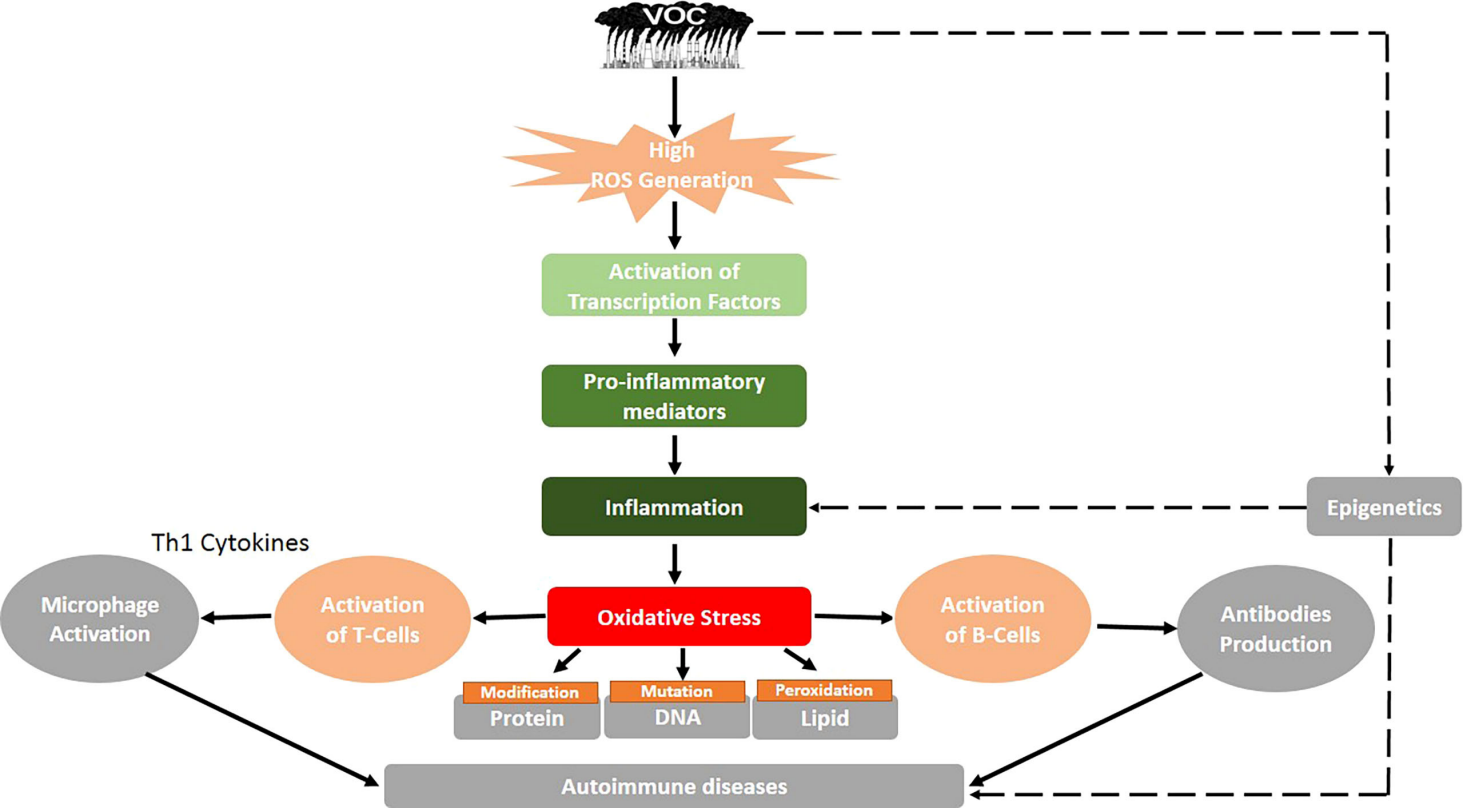
Proposed mechanism of volatile organic compound-induced inflammatory response and autoimmune diseases. Black solid arrows represent known pathways, and the black dashed arrows show an assumed mechanism, thus requiring future research.

On chronic exposure of VOCs by inhalation, oxidation stress often results due to the imbalance between the reactive oxygen species (ROS) generated and the bioavailable free radical scavengers such as antioxidants. Superoxide anion, hydrogen peroxide (H_2_O_2_), hydroxyl radical (HO^•^), and, in some cases, peroxynitrite anion are important ROS implicated in the inflammatory pathway leading to autoimmune disease (ONOO^-^) (70). Excess ROS can either oxidize biomolecules or structurally change proteins and genes, resulting in signaling cascades that can contribute to the initiation and progression of inflammatory-related diseases. ROS has been shown to be involved in the activation of redox-sensitive transcription factors such as nuclear factor kappa B (NF-kB) *via* the phosphorylation of its bound protein (IkB proteins). When activated, NF-kB dissociates from its complex and translocates from the cytosol to the nucleus, where it works alone or in synergy with other transcription factors such as AP-1, HIF-1α, PPAR-γ, and Nrf-2 to initiate the expression of specific genes involved in the synthesis of inflammatory proteins (71–74) (**Table 2**). The activation of transcription factors and pro-inflammatory genes by ROS results in the development of inflammation (**Figure 1**). The occurrence of inflammation triggers the immune cells to produce a variety of cytokines and chemokines in an attempt to recruit other immune cells such as leukocytes, polymorphonuclear neutrophils, and macrophages to the site of oxidative stress. In response, increased ROS generation by immune cells at the site of inflammation promotes oxidative stress and tissue damage and *vice versa*. Although inflammation causes oxidant injury, the reverse response is possible since inflammation and oxidative stress are mutually causal (76). Studies show that the pathogenic autoreactive antibodies/autoreactive T cells against ROS-induced VOC stays in the blood for years before the development of the active disease, making it a chronic condition if not detected at the preclinical stage (63). The various redox-sensitive transcription factors and inflammatory mediators involved in VOC-induced inflammatory response and their functions are summarized in **Table 2**.

**Table 2:** Transcription factors and inflammatory mediators involved in inflammation and oxidative stress.

Transcription factors/inflammatory mediators	Biological roles	References
Transcription factors		
Nuclear factor-kappa B (NF-κB)	Initiates the expression of inflammatory genes, inflammasome regulation, innate immune cells and inflammatory T cell regulation, and differentiation	(75)
Activator protein 1 (AP-1)	Initiates cell differentiation, survival, apoptosis, and cytokine expression	(76)
Hypoxia-inducible factor 1-alpha (HIF-1α)	Induces host immune function, vascularization, angiogenesis, cell migration, and tumor invasion	(77, 78)
Nuclear factor erythroid 2-related factor 2 (Nrf-2)	Induce the expression of antioxidant enzymes in response to oxidant exposure; inhibits inflammation	(79)
Tumor protein p53	Controls cell division and apoptosis, tumor suppression, and DNA repair	(80)
Peroxisome proliferator-activated receptor gamma (PPAR-γ)	Adipocyte regulation; blocks the expression of inflammatory cytokines and initiates immune cell differentiation	(81)
Cytokines		
Tumor necrosis factor (TNF-α and TNF-β)	Acts as an amplifier of inflammation; recruits neutrophils and macrophages	(76, 82)
Interleukins	Pro-inflammatory and anti-inflammatory; promotes activation and differentiation of immune cells, including proliferation, migration, and adhesion	(83)
Transforming growth factors (TGF-α and TGF-β)	Regulates immune cells; inhibits growth and activation	(76)
Chemokines		
Monocyte chemoattractant protein (MCP-1 and -3)	Promotes monocyte migration	(76)
Macrophage inflammatory protein-1 (MIP-1α)	Macrophage activation; recruitment of leucocytes to the site of inflammation	(84)
RANTES (regulated on activation, normal T-expressed and secreted, CCL5)	Immune cell migration	(76)

Experimental results employing a mouse model revealed that gaseous formaldehyde or low molecular weight respiratory sensitizers increase allergen-specific blood IgE or IL-4 levels, respectively (85, 86). A similar pro-inflammatory cytokine pattern with elevated levels of IL-6 or tumor necrosis factor-alpha was detected in the blood samples of children after interior remodeling activities, particularly in the presence of a new floor covering (87). These findings point to a direct interaction of VOCs with redox-sensitive pathways, which might result in a different immunological response. Several attempts have been made in the past to establish a direct link between indoor VOC exposure and the development of respiratory or allergy symptoms (88).

A mouse asthma model was directly exposed to VOCs evaporated by new PVC flooring, and their effects on the allergic immune response were monitored. Further research was conducted to determine the released VOCs as well as the observed effects of each VOC on the development of asthma in mice (68). It was revealed that PVC flooring emits a range of VOCs and that exposing mice to PVC flooring as well as to the identified single VOC increased allergic airway inflammation. The findings suggest that VOC exposure has an adjuvant effect, which is achieved through changing the Th1/Th2 balance through direct impacts on interleukin 12 (IL-12) production in dendritic cells (DCs) and causing oxidative stress in the airways (68). In summary, the cumulative effects of VOCs in living organisms may result in an increase in ROS production, which leads to inflammatory responses, the production of anti-HSP90 autoantibodies *via* HSP90 activation, cellular proliferation of Th (1, 2, 3, and 17), which leads to apoptosis, Th17 activation, and cytokine production (IL-17 and IL-23) *via* the induction of protein or lipid adducts, and modification of DNA methylation, resulting in changes in gene expression (89). **Figure 1** shows a summary of the potential mechanism of VOC-induced inflammatory response and autoimmune diseases.

## Volatile organic compounds and autoimmune diseases

**Figure 2:**
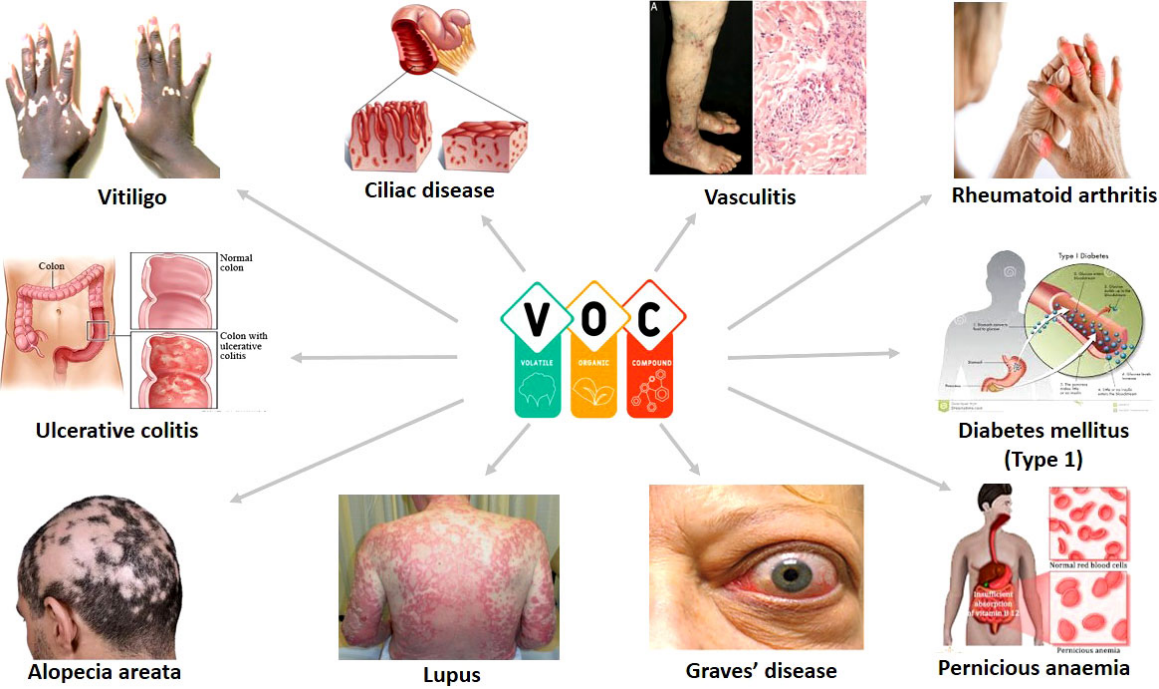
Common autoimmune diseases associated with VOC exposure.

Autoimmune diseases are caused by a lack of immune tolerance and are mediated by T or B cell activation, which causes tissue damage (**Figure 1**). The activation of T cells involves the interaction between DCs and T cell-presenting self-antigen, identification of complexes, and formation of an immunological synapse (90). The professional antigen-presenting cells (DCs) induce the differentiation of naïve CD4^+^ T cells into helper and effector T cells, causing the secretion of cytokines such as IL-12 and IL-23 (91). These cytokines direct the migration of T cells to lymphoid organs or tissues where they form regulatory T (Treg) cells. The DCs play an immunological role in the initiation and development of response and tolerogenic by promoting the imbalance between Treg cells and Th (1, 2, and 17) (92). Thus, the abnormal activation or overactivation of immune cells as a result of environmental factors such as VOCs could result in the emergence of autoimmune diseases (93). However, the immune responses triggered during disease pathogenesis follow distinct pathways. There are over 100 autoimmune diseases that can affect the human body due to VOC exposure. **Figure 2** depicts the common autoimmune diseases associated with VOC exposure.

Autoimmune diseases share common clinical signs and symptoms, physiopathological processes, and hereditary variables (94). They are multifaceted diseases produced by the long-term interplay of genetic, epigenetic, and environmental variables (95). Despite the difficulties in identifying specific environmental risk factors that contribute to the immunopathology of autoimmune disease, emerging research evidence implicates infectious agents, chemicals, physical factors, adjuvants, and hormones (96, 97). Although genetic predisposition is essential in the development of autoimmune diseases, the concordance rates among monozygotic twins were low, indicating that environmental variables play a significant role in the etiology of autoimmune diseases (98, 99).

The specific processes behind the development of environmentally caused autoimmune illnesses due to VOCs remain unclear, although several explanations have been presented for the onset of autoimmune disorders following diverse environmental exposures, including VOCs. However, none of the concepts is totally supported by direct causal evidence. Furthermore, the processes assumed to be involved in the onset of the disease process may differ from those that impair an existing condition (100). Systemic sclerosis, rheumatoid arthritis, systemic lupus erythematosus, small vessel vasculitis, and Sjögren’s syndrome are all autoimmune rheumatic disorders with minimal understanding of environmental risk factors (95, 98). However, various experimental studies and recent epidemiological data have identified certain environmental chemicals that have a similar toxicity route and mechanisms or are individually involved in the breakdown of tolerance leading to autoimmunity (95, 100).

Growing evidence indicates a role for environmental factors in autoimmune disease etiology, with the strongest evidence for smoking and occupational exposure to respirable silica dust, which has been associated with systemic autoimmune diseases, including rheumatoid arthritis (RA) and a related autoimmune disease, systemic lupus erythematosus (SLE) (98). Studies have suggested associations of SLE with occupational exposures to solvents, sun, farming, and pesticide use (101) as well as smoking (102). In the Agricultural Health Study, factors associated with incidents of RA among female farmers were investigated. Out of the 15 pesticides tested, maneb/mancozeb and glyphosate were implicated in the development of RA disease. Other chemicals such as chemical fertilizers and cleaning solvents were also associated with RA (103).

**Figure 3:**
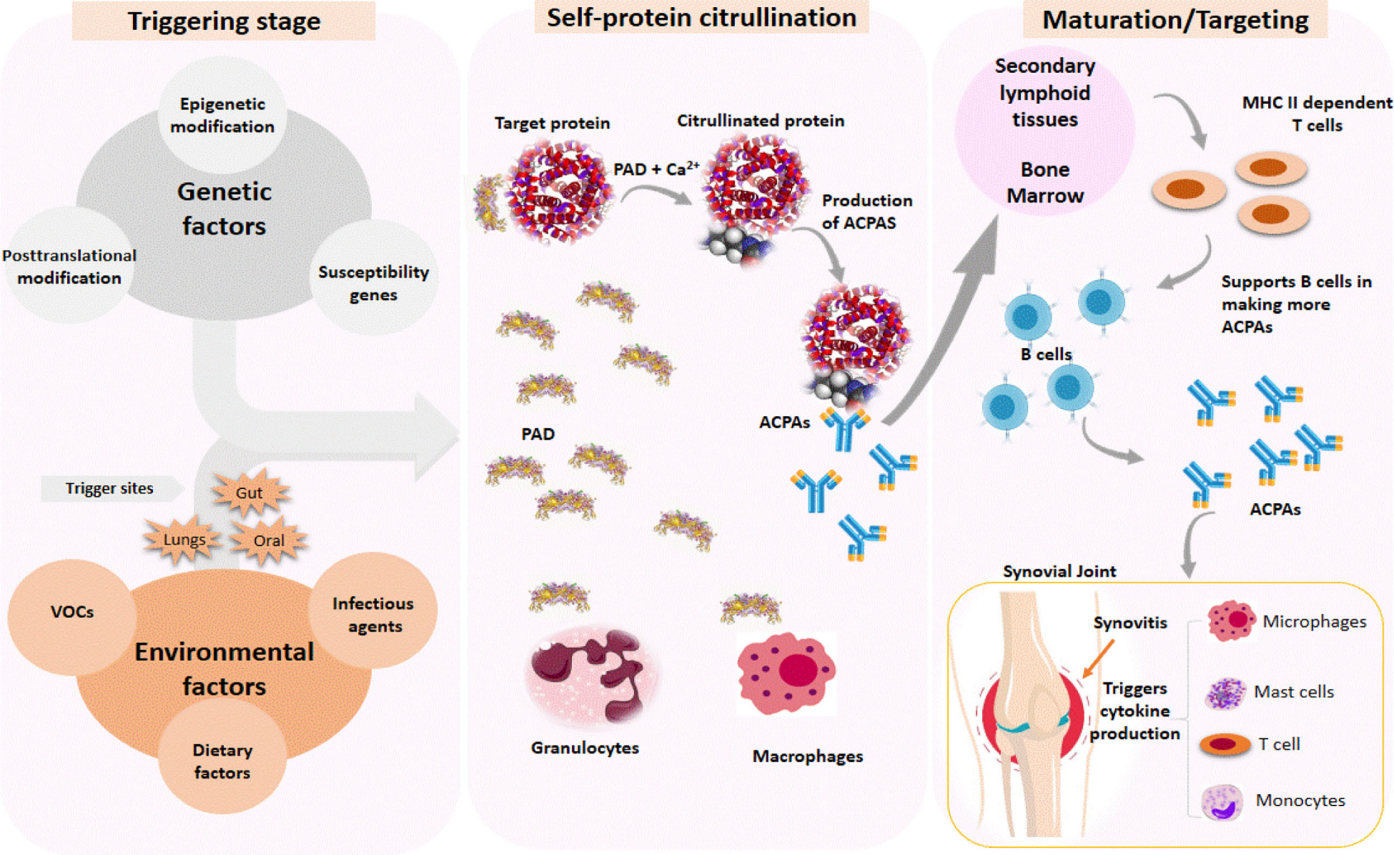
Immune response is triggered by genetic and environmental factors in the pathogenesis of rheumatoid arthritis (RA). The triggering process causes citrullination of target proteins found throughout the body, which results in the production of autoantibodies (ACPA) against citrullinated neoantigens. Increased levels of citrullinated neoantigens activate MHC class-II-dependent T cells to support B cells in producing more ACPA, which trigger cytokine production by recruiting other immune cells to the synovial joint, resulting in RA.

The triggering step in the pathogenesis of RA disease caused by VOC exposure can occur in the mouth cavities, gut, and lungs, depending on the route of exposure. The triggering process causes macrophages and granulocytes to secrete protein arginine deiminases (PAD), which require Ca^2+^ as a cofactor (**Figure 3**). PAD catalyzes the citrullination of proteins such as histones, fibrin, Epstein–Barr nuclear antigen 1, vimentin, α-enolase, and type II collagen (104). The initiation of posttranslational modification (citrullination) by PAD causes the secretion of anti-citrullinated protein antibodies (ACPAs), which target citrullinated neoantigens found throughout the body. ACPAs are produced in response to an abnormal immune response to citrullinated neoantigens. Due to its high specificity, ACPAs are currently used as a biomarker to detect and diagnose RA (104). The immune response to citrullinated neoantigens with endogenous epitopes may exist for years before the symptoms appear (105). The maturation stage occurs in secondary lymphoid tissues or bone marrow. Increased levels of citrullinated neoantigens would activate MHC class II-dependent T cells, assisting B cells in producing more ACPAs. The final stage involves the stimulation of immune cells such as monocytes, macrophages, and T cells, which causes cytokine production in the synovial joints, thus resulting in RA.

**Figure 4:**
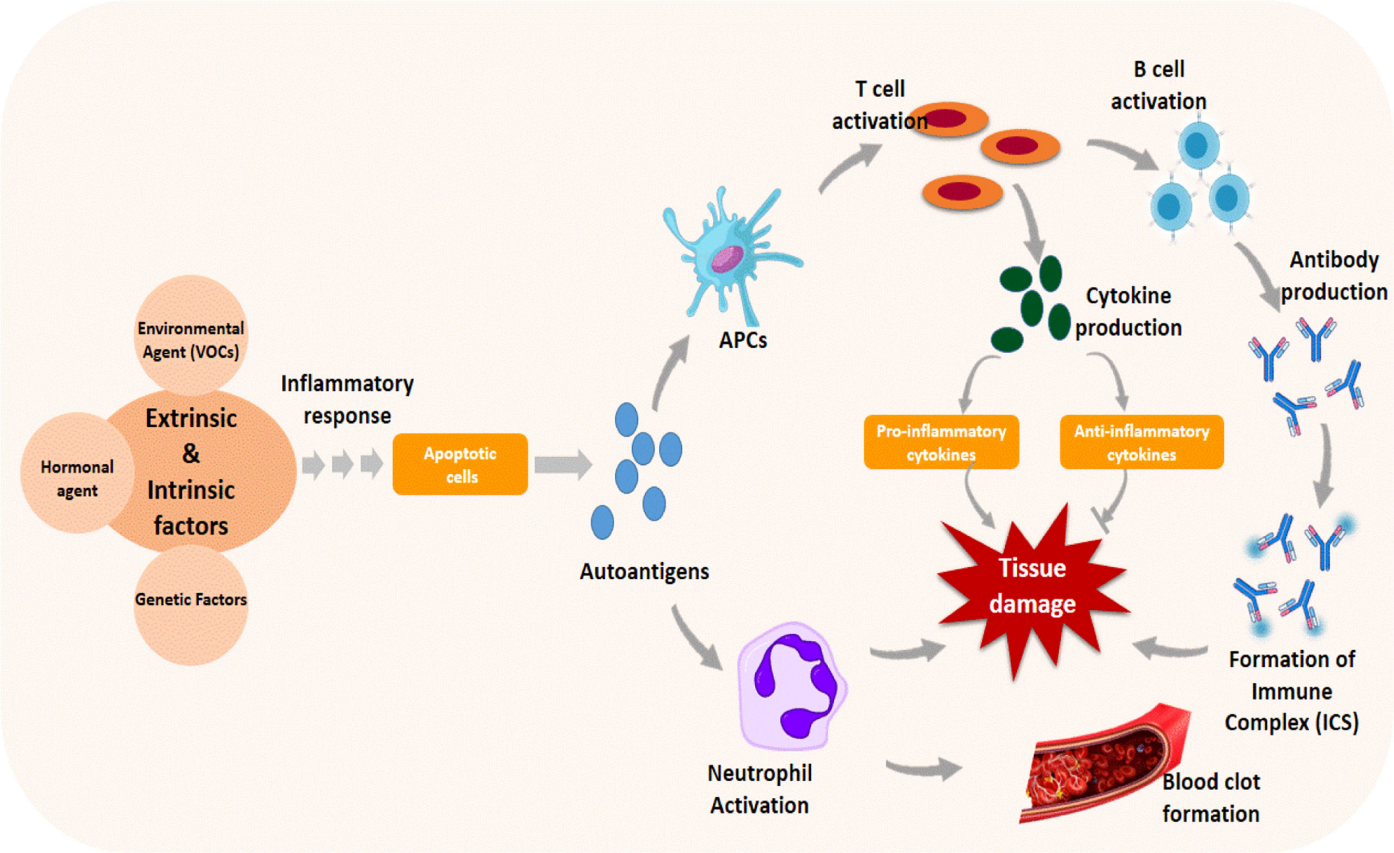
Immune response triggered by genetic and environmental factors in the pathogenesis of systemic lupus erythematosus. Apoptosis is induced by the triggering agent through cascades of cellular signaling and inflammatory responses. The apoptotic autoantigens released during the process activate antigen-presenting cells (APCs) and neutrophils, promoting thrombosis and tissue damage. Activated APC causes T cells to produce cytokines and B cells to produce other antibodies and complexes, resulting in tissue damage.

Although the actual cause of SLE is unknown, increasing research evidence suggests that a complicated interplay of genetic, hormonal, and environmental factors, such as organochlorines, trichloroethylene, and insecticides, triggers the disease’s pathogenesis (106, 107). These factors cause cell signaling and inflammatory response, which leads to programmed cell death (apoptosis) (108). Dead cells are typically removed from the body to prevent cellular complications; however, in lupus, dead cells are not removed as they should be, resulting in the disease pathology. Apoptotic cells release autoantigens, which are taken up by antigen-presenting cells, which produce cytokines and pro- or anti-inflammatory cytokines by activating T cells or may activate neutrophils, triggering a cascade of events that promote thrombus formation and tissue damage (**Figure 4**). T cells activated by autoantigens can activate B cells, resulting in the production of autoantibodies that form immune complexes (ICs) with the autoantigens. The accumulation of ICs in tissues causes the recruitment of inflammatory cells, which causes tissue damage (108).

Several household goods used on a regular basis release VOCs into the air, including some cooling and heating systems (109). These compounds disperse and recirculate in an enclosed environment, eventually settling on the scalp and hair and causing irritation and hair loss (110). Continued exposure to VOCs can cause skin inflammation (111). Air pollution can cause scalp irritation, redness, itching, excess sebum secretion, dandruff, root pain, and hair loss. The condition is known as sensitive scalp syndrome (109). The condition can mimic or overlap androgenic alopecia.

Furthermore, the risk of developing vitiligo is strongly influenced by genes passed down from parents. This is because it is more common in people whose families have vitiligo or other autoimmune diseases. A very interesting environmental factor was discovered in a large proportion of factory workers who developed vitiligo in 1939. These factory workers made leather and wore rubber gloves to protect their hands from the chemicals used in the process. However, it was discovered that the chemical in the gloves was what caused their vitiligo. The chemical is called monobenzyl ether of hydroquinone or monobenzone. In fact, it worked so well that it is now used to remove the remaining pigment from the skin of those with widespread vitiligo in order to make it even. It is prescribed by dermatologists as Benoquin cream. This incident strongly implicated chemicals as potential environmental agents that could induce vitiligo (112). The incidence of systemic sclerosis has been linked to occupational exposure to volatile organic compounds such as trichloroethylene, benzene, toluene, or xylene as well as unidentified solvents (113). With the exception of self-reports, exposure assessment primarily depended on case-by-case expert evaluation or work exposure matrices (JEMs). Self-reported job activities and JEM data have revealed links between nonspecific solvent exposure and rheumatoid arthritis, although the minimal evidence for systemic lupus erythematosus was negative (113).

VOC such as styrene has been found in a mouse model to raise the levels of interleukins (IL-4, IL-5, and IL-13) and interferon-ϒ (114). In systemic sclerosis, IL-4 and IL-13 are reported to play critical roles by differentiating T cells into T-helper type 2 (Th2) cells and inducing fibrosis (115). In addition, the Th2 response appeared to be specific for systemic sclerosis and not present for other autoimmune diseases (115–117). Based on a small number of instances, it was determined that there was a link between occupational styrene exposure and systemic sclerosis. However, it was difficult to apply this conclusion to other organic solvents since they include a diverse variety of compounds with varying toxicological characteristics.

Sjögren’s syndrome has been associated with aromatic and chlorinated solvents in a single study (118). There appears to be minimal data about particular solvents. Some studies have analyzed exposure–response relationships using semi-quantitative exposure data, but none has used quantitative exposure data (119, 120). As a result, quantifiable exposure data based on the actual measurements of individual solvents will considerably contribute to the body of information concerning the potential link between organic solvent exposure and autoimmune rheumatic diseases.

Many aromatic organic solvents, such as toluene, ethyl benzene, and xylene, are typical components of petroleum products, the majority of which have already been proven to be carcinogenic, such as benzene (121). Benzene is thought to be one of the possible reasons for morbidity among vehicle workshop workers and painters. Among many other environmental sources, benzene is primarily derived from fuel vapors and gasoline (1.8–3.7%) and solvents used for degreasing or as diluents in automotive technicians’ workspaces (122). Most organic solvent exposures occur during the maintenance of various engine parts (123). After being absorbed into the intercellular fluid, such solvents may reach the main bloodstream and spread throughout the body (124). By influencing the bone marrow, benzene has an effect on blood production. A high risk of hematological illnesses has been documented in Korean businesses as a result of comparatively high benzene exposure in the past (125). Aside from direct occupational exposure, nonoccupational populations around chemical plants and other industrial locations may also be exposed indirectly (121). Thus, occupational hematotoxicity and other blood disorders, such as blood cancer (leukemia), aplastic anemia, and dysplastic bone marrow problems, are common outcomes of benzene exposure, which can be easily screened for using full blood counts (126). Workplace exposure to VOCs and other chemicals like vinyl chloride, crystalline silica, perchloroethylene, and trichloroethylene (TCE) has been linked to a number of autoimmune diseases like lupus and multiple sclerosis (127). This is due to their ability to generate early inflammation and increase the adoptive immunological response (128).

## Genetic and epigenetic link between VOCs and autoimmune diseases

The role of genetics in the pathophysiology and in the initiation of autoimmune diseases that affects about 5–9% of the world’s population cannot be neglected. The genetic mutation leading to autoimmune disease can be a result of continuous exposure to environmental toxicants such as VOCs (129). These chemicals trigger autoimmune reactions leading to autoimmunity through direct damage to self-tissue and cause the release of autoantigens or by binding to human tissue antigens to form neoantigens (129). Research evidence shows that continuous environmental exposure to various VOCs can lead to autoimmune diseases such as lupus, rheumatoid arthritis, type 1 diabetes, Crohn’s disease, ulcerative colitis, dermatomyositis, multiple sclerosis, *etc.*, through DNA methylation (130). DNA methylation is the hub of many cellular body regulations, such as gene transcription, X-chromosome inactivation, chromosome stability, and genomic imprinting (131). These chemicals interfere with the one-carbon, citric acid metabolic pathway and enzyme activities leading to abnormal DNA methylation in the genome (132). DNA methylation alteration in common autoimmune diseases is depicted in **Table 3**.

**Table 3:** DNA methylation alterations in autoimmune disease.

Autoimmune disease	DNA methylation/alteration	VOCs linked with DNA methylation/alteration	Consequences	Examples of affected genes
Lupus	Hypomethylation	Acetonitrile, toluene, hexane, 2-methylpentane, methyl cyclopentane and 3-methylpentanetoluene, benzene, xylene, mercury, vinyl chloride, perchloroethylene, trichloroethylene, and crystalline silica	Gene activation	*CD70*, *CD154*, *IL-4*, *IL-6*, *CD9*, and *MMP9* (cytokines and signaling molecules)
Lupus	Hypermethylation	Cadmium, vinyl chloride, perchloroethylene, trichloroethylene, smoking, silica dust, pesticides, and exposure to livestock	Gene silencing	*RUNX3* (folate biosynthesis genes), *IL-2*, and *foxp3* (Treg generation)
Rheumatoid arthritis	Hypomethylation	Crystalline silica, vinyl chloride, perchloroethylene, trichloroethylene, and smoking	Gene activation	*CD40L*, *IL-6*, and *IL-1* (cytokines and signaling molecules)
Type 1 diabetes	Hypermethylation	Toluene, benzene, xylene, petroleum hydrocarbon products, fuel vapor, and gasoline	Gene silencing	Insulin and *foxp3* (Treg generation, insulin production in pancreatic cells)
Vitiligo	Hypermethylation	Monobenzyl ether of hydroquinone or monobenzone	Gene activation	*NLRP1* and *PTPN22* (regulate inflammatory response and signal transduction)
Pernicious anemia (PA)	Hypermethylation	Petroleum hydrocarbon products, fuel vapor, and gasoline	Gene activation	Many genes are affected depending on the type of PA such as *FCGR2A*, *PTPN22*, *SERPINA1*, *IL12B*, *P4HA2 PRTN3*, *etc.*
Vasculitis	Hypermethylation	New building scent and nitric acid	Gene activation	*HLA-DP* and *HLA-DQ* variants, *SERPINA1*, *PRTN3*, and *PTPN22*
Alopecia areata	Hypermethylation	Cooling and heating systems and household goods	Gene activation	*PRDX5* and *STX17* (hair follicle-specific genes)

Epigenetics can be referred to as the external modification to the DNA of an organism that turns genes on or off. These modifications do not change the DNA sequence; however, it is seen in how the cells “read” genes (133). DNA modification can occur in the form of DNA methylation. DNA methylation involves the addition of a methyl group to cytosine in the DNA sequence—specifically the ones followed by guanine to prevent the binding of transcription factors and certain genes from being expressed, leading to system malfunction (134). Environmental factors such as the generation of VOC can modify cellular processes that dictate the protein to be suppressed or activated (132). These chemicals also alter a number of CD4T cell genes (transcriptional factor) at the promotor region, triggering demethylation, transcription, and translation and leading to the production of cytokines, various T-helper cell subtypes (Th1, Th2, Th17, and Treg), adhesion molecules (CTLA-4, CD40L, and CD70), cell cycle proteins (Cdkn1a), and then autoimmune diseases (130). The various types of autoimmune diseases are viewed as collections of different disease phenotypes that arise as a result of the gene environmental interactions affecting both adaptive and innate immunity (135). The combination of the effects of gene environmental interactions leads to many phenotypic diseases such as lupus, rheumatoid arthritis, type 1 diabetes, and thyroiditis (136).

## Volatile organic compound exposure and health defects

The presence of varieties of VOCs in the environment has become a major health concern for over three decades due to the adverse health effects exerted by these pollutants. Exposure to VOC in the environmental media can be by ingestion and skin contact, and the highest concentrations is most frequently found in air (137). Although some inhaled VOCs have been shown to be absorbed in the upper respiratory tract, the majority of systemic absorption occurs in the alveoli, the inner region of the lungs. This process is facilitated by an increase in breathing and cardiac output/pulmonary blood flow, which permits VOCs to be delivered to tissues by blood *via* the arteries. The extent of their uptake in the tissues is determined by blood/air partition coefficients. As a result, extrahepatic organs receive higher dosages after inhalation exposure. After inhalation, VOCs rapidly build up in the brain, providing central nervous system (CNS) effects as deep as a surgical anesthetic in as little as 1 to 2 min. Furthermore, adipose tissue stores a considerable amount of VOCs (137).

Exposures to volatile organic compounds have been associated with a wide spectrum of diseases that affect human health, from mild illnesses such as irritation to derogatory diseases that include cancer. The adverse effects of VOCs have been seen even at low levels of exposure in several epidemiological studies. Studies have shown that diseases such as sensory irritation and respiratory symptoms, organ damage, nervous system defects, autoimmune diseases, and cancer are linked with a substantial exposure to VOCs which enter the human body through different ways such as inhalation, ingestion, and skin absorption to cause pathologies (13, 20, 138). Diseases linked with VOC exposure include pulmonary diseases, immunological system disorders, acute myeloid leukemia in children, skin diseases, and rheumatoid arthritis (20, 139, 140). VOCs also cause acute symptoms such as headaches, dizziness, irritations of the eyes, throat, and nose, allergic skin reactions, and nausea. In other cases, exposure can lead to the damage of internal organs such as the kidney and the liver. Exposure to VOCs may not always result in immediate hazards, but they are often associated with causes of chronic health disease (141).

## Non-autoimmune disease associated with volatile organic compounds and pulmonary/respiratory diseases

Pulmonary ailments and diseases of the respiratory system are one of the major causes of mortality and burden in the form of healthcare costs in the world today. Asthma, chronic obstructive pulmonary disease, and lung cancer are prevalent pulmonary diseases caused by exposure to VOCs. According to Alford and Kumar (142), high levels of reaction produced by VOCs in the airway epithelium and mucosa membrane are associated with pulmonary diseases. A total of 49 studies were carried out to measure the health outcomes caused by VOCs in high-income countries, including seven individuals from France, six individuals from the USA, and seven individuals from Japan (142). The result of their studies indicated that VOCs have a medium effect on pulmonary diseases but are implicated in the onset of asthma and wheezing. Another study conducted by Cakmak et al. (143) shows that elevated indoor levels of VOCs are linked with a decline in lung functions. Out of 10 VOCs evaluated, nine were linked to causing a significant reduction in the measure of lung function in children under 17 years of age, while three VOCs (hexanal, 2-methyl-1,3-butadiene, and α-pinene) were recorded to be the major VOCs linked with lung function decline in elders above 65 years. VOCs relatedness to respiratory health were assessed in French farmers to determine the connection between indoor exposure to VOCs and particulate matter with respiratory health. Among the VOCs evaluated, trichloroethylene and benzene showed a significant association with asthma, whereas styrene and acetaldehyde were linked with early airway obstruction and halogenated hydrocarbons showed a promoting effect on asthma incidence (144). Furthermore, VOC profiling is used in breath analysis to distinguish different forms of respiratory diseases. Smolinska et al. (145) utilized this method of VOC breath analysis profiling to discriminate preschool transient wheezing children from asthmatic ones.

## VOCs and organ dysfunction and diseases

Several volatile organic compounds are listed as high-risk health-degrading factors as a result of the direct toxic effects they have on multiple organ targets and their complex mechanism of action. Additionally, they have a high potential risk for human exposure (146) and are associated with liver and kidney damage as well as cardiovascular diseases (147). Reactive metabolites in VOCs such as aldehydes cause oxidation and protein carbonylation, which is the root of most modified protein and downstream dysfunctions that damage organelles such as the mitochondrion and cause cell damage and bioenergetic dysfunction leading to organ damage (138). A high-level exposure to VOCs shows hepatotoxicity in humans and promotes underlying injuries to liver function, thus causing liver damage and failure in severe cases. Pijls et al. (148) applied VOC analysis in a discriminatory study in a heterogeneous group of patients with liver dysfunction to differentiate patients with chronic liver disease (CLD) from patients with compensated cirrhosis (CIR) and those without cirrhosis. The study showed five serological markers to differentiate CLD patients from CIR patients at sensitivity and specificity of 0.71 and 0.84, while a set of 11 volatile organic compounds was used to distinguish CIR from CLD patients with a sensitivity of 0.83 and specificity of 0.87, indicating that exposure to VOCs is linked to liver dysfunction and diseases. More recently, Sinha et al. (149) analyzed the interrelatedness between VOCs and non-alcoholic fatty liver disease (NAFLD) using VOC profiling from breath analysis. In the study, volatomics was used to discriminate healthy individuals from patients with NAFLD cirrhosis and individuals with NAFLD cirrhosis from patients with non-cirrhotic NAFLD. Indications from the study showed that terpinene, D-limonene, and dimethyl sulfide from breath analysis are potential volatomic markers that show the linkage between VOC exposure and non-alcoholic fatty liver disease. In addition to the hepatotoxicity effects, VOCs are known to impede renal functions in humans and animals. The thin-film transistor-liquid crystal display (TFT-LCD) is an emerging industry worldwide that has been implicated as a high-risk job that exposes workers to VOCs that lead to a high rate of kidney dysfunction. This is a result of the prevalence of VOCs such as ethanol, isopropyl alcohol, and acetone which are used as solvents in the industry. The findings of Chang et al. (150) revealed that an array of workers in the TFT-LCD industry are at a high risk of developing kidney disease and that VOCs are highly associated with renal dysfunction. Furthermore, Obermeier et al. (151) employed VOC profiling from breath analysis as a non-invasive technique for discriminating children with chronic kidney disease (CKD) from healthy ones. Tests have shown that volatile compounds that included ammonia, isoprene, pentanal, ethanol, heptanal, and methylamine were found in higher concentrations in CKD patients than in healthy children.

## VOCs and nervous system damage

Volatile organic compounds present in the air as pollutants have been associated with diseases that affect the CNS, such as Alzheimer’s disease, Parkinson’s disease, stroke, and other neurodevelopmental disorders. Various research records have shown that VOCs in the blood can easily move to the CNS and activate responses that result in nervous system disorders. Additionally, the effects of VOCs on other systems, such as the pulmonary and cardiovascular systems, can affect the health of the CNS (152). Several VOCs are neurotoxic, causing neurological pathology and neurocognitive impairment through oxidative stress, neuroinflammation, glial activation, and cerebrovascular damage (13, 152). Benzene, toluene, ethylbenzene, and xylene are known VOCs that affect the central nervous system alongside the reproductive and immune systems in humans (153, 154). More so, VOC exposure has been linked with an elevated risk of the neural tubes, while acrylamide is associated with damages of the central and the peripheral nervous systems (13, 155).

## VOCs and cancer

Several VOCs have been identified to have mutagenic and carcinogenetic activities. The International Agency for Research on Cancer has identified some VOCs—benzene, trichloroethylene, 1,3-butadiene, crotonaldehyde, xylene, acrolein, and toluene—as carcinogenic compounds. More so, compounds such as N, N-dimethylformamide, ethylbenzene, acrylamide, and acrylonitrile have been classified as probable carcinogens (13, 156).

Current investigations in cancer research utilize noninvasive techniques that employ VOC profiling from breath, fecal, and urinal analysis in discriminatory cancer research. This is due to the interrelatedness of VOC exposure with cancer incidence. Heptanal, nonanal, decanal, and hexanal are VOCs classified under the aldehydes that are linked with cancerous cell growths. Among the aldehydes, heptanal has been related to eight different cancer types. Other VOCs that have been linked to cancer and are utilized as biomarkers for early the detection and diagnosis of cancer in human cells include ketones such as cyclohexanone, 2-nonanon, 3-heptanone, and 4-hepatnone (157).

## Preventive strategies against VOC exposure

Following the negative implications and impact of exposure to VOCs, preventive strategies against exposure to VOCs are important to guarantee a healthy lifestyle. Addressing potential exposure around one’s comfort zone, for instance, at home or a space wherein one spends a lot of time, can ensure that maximum health safety is reached. Possible routes of exposure must be considered and addressed to maintain safety around oneself. Known routes of exposure to VOCs include internal and external sources. Some internal sources of exposure include exposure at home and in comfort zones wherein VOCs can be found in items such as cleaning products, cosmetics, energy generation supplies (such as fuel, gas, oil, and wood burning), adhesives, paints, and solvents among other sources. Some external sources can be found outdoors, with sources such as wood burning, factory/industrial processing and manufacturing works, gas emissions, gas extraction, and processing among other sources.

The effects of VOC exposure are dependent on many factors, some of which include the means or route of exposure, type of VOC, duration of exposure, and the exposed person (158). The route of exposure considers the form following the contact with one, and this determines potential harm or not to the exposed person. The type of VOC considers the source and form of the organic compound as some may pose minimal to insignificant harm, whereas many may pose harmful long-term effects on the exposed person (MDH), 2022). The duration of exposure focuses on the frequency and timeline following exposure and how much damage may occur within the stated period. The exposed person simply considers the reaction pattern of one exposed to the VOC since some people may react badly to some VOCs or have deadly allergic triggers/health implications unlike some other persons, as there will be variations in different reaction forms by different individuals. A typical example is the fact that an asthmatic person, for instance, may have a more terrifying reaction to some VOCs than a supposed normal person who may not react (ALA, 2020). These factors do not negate the fact that there are some compounds (VOCs) that, upon exposure even in minimal forms, will cause harmful effects on any and every human, many of which will cause long-term effects (ALA 2020).

While it is almost impossible for humans to completely avoid VOCs, adequate care must be ensured to prevent frequent/prolonged exposure, and since VOCs are volatile and many are unstable, exposure can be highly risky healthwise. Some preventive strategies to ensure the proper use of and prevent exposure to VOCs include following instructions on product labels correctly, ensuring the proper storage of products, and regular adoption and revision of safety control protocols (159, 160). Proper ventilation practices should be adopted. Ensure new products are allowed to air out before taking indoors to release VOCs (161). Keep the products out of reach of children, and discard any remaining products that may not be used again.

Limiting exposure to VOC is the ultimate goal with regard to protecting one’s health, for instance, opt for VOC-free products for homes, workplaces, and the environment (159). Regulate the in-house temperature to prevent activating some chemicals at home to give off gas which usually occur at high temperatures or upon exposure to heat (158). Ensuring adequate ventilation and using air purifiers around one’s home or workplace can help limit the exposure. The use of air purifiers can be adopted to eliminate VOCs indoors or at home. Purifiers with carbon filters, for instance, work by eliminating gaseous VOCs from air circulating around the home following an adsorption process that the filter uses by adsorbing the pollutants present (162). Photo-electrochemical oxidation purifiers attract VOCs present and destroy them using light-activated catalysts which operate by attracting the VOCs with its filter; hydroxyl radicals are generated which react with the VOCs in the air and destroy them (162). In situations where applicable, one should choose alternative options from high-VOC ones, thereby minimizing the prospects for exposure. An example is with cleaning agents: one can choose low-VOC agents over the “regular” ones (159).

Ensuring that the safety protocols and instructions are adhered to is important to limit exposure to VOCs (159). Environmental safety tests should be performed at homes and workplaces to ensure that there is adequate safety for humans in the environment. The use of natural products should be adopted where possible. Ventilation is necessary in the home and outside, especially when working with any of the chemicals. Avoid exposure to smoke. Training and workshops to guide housemates and employees should be encouraged to minimize exposure and risks following the use of VOCs (159).

## The way forward: management of VOC-induced health effects

Prevention and limiting/minimizing exposure are the best approaches towards ensuring that one has a healthy life ahead (163). The first hand of treatment or management should be the removal of the contaminating source of VOC, after which immediate symptoms may improve. VOC removal can occur following the use of nano-materials with techniques such as catalysis and adsorption (26). Oftentimes, management/treatment strategies and patterns depend on the symptoms presented following an exposure. VOC exposure symptoms range from milder ones like skin irritation or headaches to more serious symptoms such as central nervous system toxicity and cancer (164)—for instance, a child or an adult who presents with wheezing symptoms or breathlessness (difficulty breathing) will have a physician treat the presented symptom accordingly to ease the discomfort as fast as possible while considering the long-term possible effects of exposure to the VOC which triggered the uncomfortable symptoms. Persons exposed to VOCs should be monitored both on a short- and a long-term bases.

## Conclusion

Major emphasis should be directed on occupational exposure to VOC and other sources of toxicants in the workplace, which are the root causes of pathophysiological changes due to accumulated effects years before the onset of several autoimmune diseases. There is a need for total adherence to safety rules such as the use of personal protective equipment and sitting of industrials far away from residential buildings to reduce the effects of these hazardous chemicals. Environmental protection should be taken seriously because the excess of these toxicants may help in promoting autoimmunity through alterations in DNA methylation in CD4 T cells.

Abbreviations

VOCs, volatile organic compounds; TNF-α, tumor necrosis factor alpha; IL-6, interlukin-6; IL-1, interlukin-1; IFN-γ, interferon gamma; NO*_x_*, nitrogen oxide; O_3_, ozone; IgE, immunoglobin E; PG, propylene glycol; EPA, Environmental Protection Agency; VVOCs, very volatile organic compounds; SVOCs, semi-volatile organic compounds; PCBs, polychlorinated biphenyls; PAHs, polycyclic aromatic hydrocarbons; PBDEs, polybrominated diphenyl ethers; PAEs, phthalic acid esters; PMF, positive matrix factorization; BVOCs, biogenic volatile organic compounds; MQL, method quantification limit; PC, partition coefficients; CNS, central nervous system; ROS, reactive oxygen species; ADs, autoimmune diseases; COPD, chronic obstructive pulmonary disease; CLD, chronic liver disease; CIR, compensated cirrhosis; NAFLD, non-alcoholic fatty liver disease; TFT-LCD, thin-film transistor-liquid crystal display; CKD, chronic kidney disease; BTEX, benzene, toluene, ethylbenzene, and xylene; IARC, International Agency for Research on Cancer

## Author contributions

The manuscript was written collaboratively by all of the authors including review/editing processes. All authors approved the submitted version.

## Conflict of interest

The authors declare that the research was conducted in the absence of any commercial or financial relationships that could be construed as a potential conflict of interest.

## Publisher’s note

All claims expressed in this article are solely those of the authors and do not necessarily represent those of their affiliated organizations, or those of the publisher, the editors and the reviewers. Any product that may be evaluated in this article, or claim that may be made by its manufacturer, is not guaranteed or endorsed by the publisher.
